# Drug Repurposing for Targeting Myeloid-Derived Suppressor-Cell-Generated Immunosuppression in Ovarian Cancer: A Literature Review of Potential Candidates

**DOI:** 10.3390/pharmaceutics15071792

**Published:** 2023-06-22

**Authors:** Yani Berckmans, Yannick Hoffert, Ann Vankerckhoven, Erwin Dreesen, An Coosemans

**Affiliations:** 1 Laboratory of Tumor Immunology and Immunotherapy, Department of Oncology, Leuven Cancer Institute, Katholieke Universiteit Leuven, 3000 Leuven, Belgium; 2 Clinical Pharmacology and Pharmacotherapy Unit, Department of Pharmaceutical and Pharmacological Sciences, Katholieke Universiteit Leuven, 3000 Leuven, Belgiumerwin.dreesen@kuleuven.be (E.D.)

**Keywords:** ovarian cancer, drug repurposing, myeloid-derived suppressor cells, treatment strategies

## Abstract

The lethality of patients with ovarian cancer (OC) remains high. Current treatment strategies often do not lead to the desired outcome due to the development of therapy resistance, resulting in high relapse rates. Additionally, clinical trials testing immunotherapy against OC have failed to reach significant results to date. The OC tumor microenvironment and specifically myeloid-derived suppressor cells (MDSC) are known to generate immunosuppression and inhibit the anti-tumor immune response following immunotherapy treatment. Our review aims to characterize potential candidate treatments to target MDSC in OC through drug-repurposing. A literature search identified repurposable compounds with evidence of their suppressing the effect of MDSC. A total of seventeen compounds were withheld, of which four were considered the most promising. Lurbinectedin, metformin, celecoxib, and 5-azacytidine have reported preclinical effects on MDSC and clinical evidence in OC. They have all been approved for a different indication, characterizing them as the most promising candidates for repurposing to treat patients with OC.

## 1. Introduction

Ovarian cancer (OC) is the fifth leading cause of cancer-related deaths worldwide [1]. This poor prognosis is mainly ascribed to the most prevalent subtype, high-grade serous epithelial OC (HGSOC), with a 5-year survival rate of just 29% [2,3]. Other epithelial OC included low-grade serous OC, endometroid, mucinous and clear-cell OC. Non-epithelial OC accounts for only 5–10% of all OC [1]. HGSOC, the main focus of this review, has the reputation of being a ‘silent killer’ due to the seemingly harmless, vague symptoms resulting in the disease being diagnosed at stage III or IV in 70% of patients [1,4,5]. At these advanced stages, the most distinctive symptom is the buildup of ascites resulting in abdominal pain and distention, which can ultimately lead to respiratory and gastrointestinal problems [6,7]. The standard treatment of HGSOC consists of cytoreductive debulking surgery and platinum-based chemotherapy. However, relapses are frequent (>80%) and often accompanied by the development of platinum resistance [6]. Despite the introduction of the monoclonal antibody (mAb) bevacizumab (angiogenesis inhibitor) and poly(ADP-ribose) polymerase (PARP) inhibitors, only a marginal improvement in the prognosis of OC has been observed in recent decades and mortality rates continue to remain high [6,8,9].

Recently, there has been a growing interest in the development of immunotherapy, especially immune checkpoint inhibitors (ICI), for the treatment of OC [10]. However, multiple clinical trials testing ICI treatment in HGSOC patients have failed to show significant results so far, even in combination with chemotherapy [11,12,13]. A possible explanation for these sobering results can be associated with the strong immunosuppression present in the immune microenvironment [14]. Specifically for OC, one major driver of this immunosuppression arises from the recruitment of tumor-stimulating immunosuppressive cells such as tumor-associated macrophages (TAM), myeloid-derived suppressor cells (MDSC), and regulatory T-cells (Treg) [15,16]. The occurrence of these cells can be correlated with a reduced clinical outcome [17,18,19]. The enrichment of MDSC, which is the main focus of this review, has been particularly associated with a decrease in progression-free survival rates in HGSOC patients [20,21]. However, current immunotherapy approaches are not focused on targeting these cells.

MDSC are a heterogeneous population of mostly immature myeloid progenitor cells with immunosuppressive functions [22]. Multiple mechanisms have been identified through which MDSC can suppress the anti-tumor immune response. MDSC can suppress the anti-tumor immune reaction through direct cell–cell interaction, mediated by the binding of ligands and receptor proteins on the surface of the immune cells [23]. Examples of such ligand–receptor interactions are programmed cell death ligand-1 (PD-L1) and its receptor programmed cell death protein-1 (PD-1) [24] as well as a disintegrin and metalloproteinase domain 17 (ADAM17) and its receptor CD62L [25]. Secondly, MDSC can suppress the anti-tumor immune response through the production of immune modulatory factors such as arginase-1 and indoleamine-2,3 dioxygenase, which can cause the depletion of essential metabolites critical to standard immune functioning (L-arginine and L-tryptophan, respectively) [26,27]. Additional factors produced by MDSC, such as reactive oxygen species and nitric oxide, can further impair the anti-tumor immune function and even be toxic to the surrounding cells [28,29]. Moreover, MDSC can indirectly inhibit the immune response through the production of cytokines such as interleukin (IL)-10 and transforming growth factor-beta (TGF-beta) [27,30]. Finally, MDSC can secrete soluble factors such as matrix metalloproteinases, vascular endothelial growth factor, and TGF-beta, which stimulate non-immunological processes such as metastases, neovascularization, and tumor growth [26,27].

By definition, drug repurposing refers to the use of established therapeutics for treatments outside the scope of the original drug indication [31]. A major advantage is the lower attrition rate due to safety and feasibility issues, as the previously accumulated toxicological and pharmacological evidence suggests sufficient safety and feasibility in human administration. Furthermore, the cost and time needed for the development are lower, as most of the preclinical (safety) testing and formulation development has been completed [32,33]. This results in a reduced risk of failure of the repurposed drug, especially concerning the safety profile [34]. Additionally, the lower risk and more rapid return on the investment show promise for pharmaceutical companies looking into this type of drug development [33]. The most well-known example of drug repurposing outside the oncology field is sildenafil citrate, which was originally developed for the treatment of hypertension but failed to show improvements. Later, it was rescued and rebranded as Viagra™ for the treatment of erectile dysfunction [31,32]. In oncology, drug repurposing has also been applied to drugs such as doxorubicin [34] and all-trans retinoic acid (ATRA) [31]. With this objective, databases such as the ReDO database [31,35], DeSigN [36], IMPACT [37], and DRUGSURV [38] were created, gathering all drug candidates to facilitate the drug development process. More recently, model-informed drug repurposing (MIDR) strategies were proposed to streamline the drug repurposing process [39]. During the COVID-19 pandemic, MIDR approaches were extensively used to characterize and screen potential candidates against the novel SARS-CoV-2 virus [40]. Nevertheless, briskly conducted trials with several approved compounds testing single doses often resulted in the termination of trials due to inefficacy. Although the repurposed compounds were well characterized with an in-depth knowledge of their pharmacokinetics (PK), the exposure–response rationale was often overlooked during drug repurposing. Nevertheless, MIDR based on pharmacokinetics–pharmacodynamics (PKPD) models can guide the search for doses by simulating and exploring the dose–exposure–response relationship and providing an informed solution to address this problem [40].

Because of the advantages of the drug repurposing process, the present review summarizes possible candidate drugs for repurposing, aiming to target the different immunosuppressive and tumor-promoting functions of MDSC in the tumor microenvironment (TME) of OC and the representative available evidence. The review builds upon extensive literature research and aims to shed light on possible candidates, addressing MDSC immunosuppression and aiming to improve combinational treatment for patients with OC.

## 2. Materials and Methods

A literature search and subsequent screening procedure were performed by examining the PubMed database. The search term included all synonyms and MeSH terms for OC and MDSC, which was concluded to the full search term depicted in Supplementary Materials S1. The final search was conducted on the 26 April 2022. Articles were independently reviewed by two authors (Y.B. and A.V.) based on title and abstract using the Rayyan platform [41]. Inclusion criteria consisted of (i) articles in English, (ii) preclinical research in OC, (iii) testing compounds already approved for human use or approved for clinical testing for other indications or OC, and (iv) reported effects on myeloid cell compartment after compound administration. A standardized data-collection approach was used to extract relevant information, including the compound, the type of combinational treatment, the effect on MDSC, and the investigated research setting, such as in vitro, in vivo, or in silico.

## 3. Results

**Figure 1:**
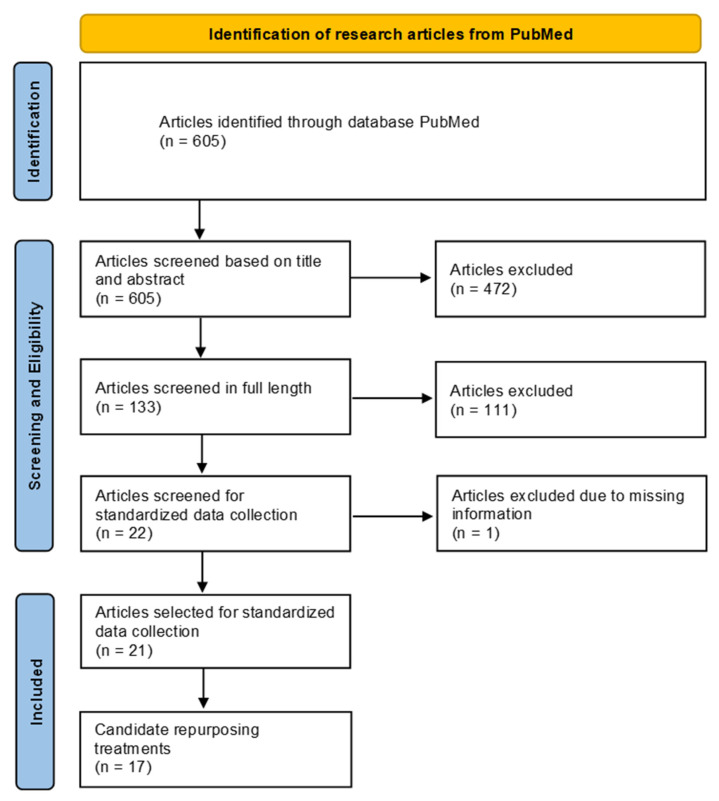
PRISMA figure depicting the strategy and screening procedure for the literature search.

The screening procedure and results are depicted in the PRISMA flow diagram (Figure 1). The complete search term retrieved 605 matching articles on PubMed. A first screening based on title and abstract resulted in the exclusion of 472 articles due to either being the wrong study type or not including the investigation of myeloid effects. The remaining 133 full-length articles were further screened, after which 22 articles were selected for standardized data collection. The other 111 articles were excluded because they described a compound with no reported experience of administration in humans. Later, one article was excluded due to missing information. From the standard data screening, 17 compounds or possible treatment combinations were selected as candidates for repurposing based on their beneficial effects on MDSC immunosuppression, as shown in OC research. An overview of the compounds and their structure is depicted in Supplementary Figure S1.

**Figure 2:**
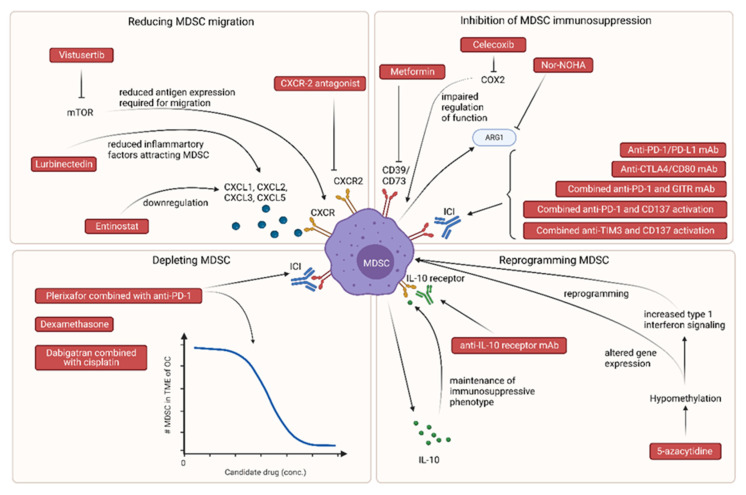
Overview of drug candidates for repurposing and their effect on myeloid-derived suppressor cells (MDSC). ARG1, Arginase 1; COX2, cyclooxygenase 2; CTLA4, CTLA4 cytotoxic T-lymphocyte associated protein 4; CXCL, C-X-X chemokine ligand; CXCR, C-X-X chemokine receptor; ICI, immune checkpoint inhibitors; IL-10, interleukin 10; MDSC, myeloid-derived suppressor cells; mTOR, mammalian target of rapamycin; OC, ovarian cancer; PD-1, programmed cell death protein 1; TIM3, T cell immunoglobulin and mucin-domain-containing protein 3; TME, tumor microenvironment; #, number. Made in Biorender.com.

As MDSC can be targeted in multiple ways, all candidates were divided based on their strategy of MDSC inhibition. This division was based on and adapted from a review by Wesolowski et al. [42], consisting of (1) the reprogramming of MDSC, (2) reducing MDSC migration to the TME, (3) MDSC depletion, and (4) the inhibition of MDSC immunosuppressive functions. Figure 2 provides an overview of all 17 candidate drugs or drug combinations and their potential influence on MDSC in OC.

### 3.1. Candidate Treatments Reducing MDSC Migration to the Tumor Microenvironment

#### 3.1.1. CXCR-2 Antagonists

C-X-C chemokine receptor 2 (CXCR2)-antagonists such as AZD5069 or SB265610 are small-molecule, reversible, allosteric antagonists at the human CXCR2 receptor, preventing receptor activation [43]. The specific antagonist AZD5069 has been tested in clinical trials for the treatment of lung diseases such as asthma, chronic obstructive pulmonary disease, and bronchiectasis [44]. Its safety and efficacy were investigated in patients with severe asthma (NCT01704495). While the treatment was well tolerated in all dose groups tested (twice daily oral administration for six months), no improved clinical outcome was observed in these patients. The most commonly reported adverse event in these patients was nasopharyngitis [44]. Despite this, AZD5069 was granted a pediatric investigation plan by the European Medicine Agency (EMA) for the treatment of uncontrollable asthma through the oral administration of tablets in patients from 6 to 18 years of age with a history of exacerbations [45]. Moreover, in metastatic castration-resistant prostate cancer patients, AZD5069 showed anti-tumor activity in combination with enzalutamide [46]. Interestingly, CXCR2 is expressed on MDSC. This is the major receptor involved in MDSC infiltration to the tumor site after binding with its ligand, CXCL1 or CXCL2, produced by the tumor cells. The antagonist SB265610 suppressed MDSC migration to the tumor and delayed tumor growth in OC-bearing mice when administering a dose of 2 mg/kg bodyweight [47]. In OC patients, the overexpression of CXCR2 was correlated with low overall survival in patients with primary epithelial OC [48]. However, to date, no clinical trials were conducted on OC to test the inhibition or blocking of this receptor using an antagonist.

#### 3.1.2. Entinostat

Entinostat, a class-1 histone deacetylase inhibitor, was first approved in 2010 as an orphan designation for the treatment of Hodgkin’s lymphoma [49]. Additionally, entinostat is currently investigated in different phases of clinical trials because of its antineoplastic activity toward various cancer cell lines [50]. Side effects can occur and include fatigue, nausea, vomiting, diarrhea, hematologic toxicity (anemia, neutropenia, thrombocytopenia), dyspnea, peripheral edema, and decreased weight [51]. In a study of its effect in OC, performed by McCaw et al., entinostat was administered in multiple intraperitoneal doses between 20 and 50 mg/kg bodyweight. The treatment of OC-bearing mice resulted in the upregulation of pathways involved in effector CD8+ T-cell functioning. Furthermore, it also caused a depletion of MDSC in the TME, which resulted from the downregulation of chemoattractant molecules such as CXCL1, CXCL2, CXCL3, and CXCL5 [52]. A phase 2 trial (NCT02915523) investigated efficacy and adverse events as a measure of safety and tolerability for patients with epithelial OC, taking 5 mg entinostat orally once a week in combination with a checkpoint inhibitor, avelumab. This study showed increased toxicity in the combination-treated group without an improvement in progression-free survival compared to avelumab alone [53].

#### 3.1.3. Vistusertib

Vistusertib, or AZD2014, is an orally available, specific dual-mTOR kinase inhibitor (mTORC1 and mTORC2) [54]. Inhibition of the mTOR pathway may result in the induction of the apoptosis of tumor cells. This mechanism of action is evaluated for the treatment of several cancer types, including breast cancer, non-small cell lung cancer, and adenocarcinoma. Reported side effects include fatigue, gastrointestinal symptoms, and rash [54]. In OC, preclinical evidence was presented in an article by Pi et al., where they tested the effect of the oral administration of 25 mg/kg bodyweight on mice. The treatment with vistusertib showed reduced ascites and prolonged survival in mice [55]. Others have reported the importance of the mTOR signaling for the recruitment of MDSC to the TME by modulating their antigen expression [56]. In line with this, Pi and colleagues found a diminished MDSC population in the peritoneal ascites fluid of OC-bearing mice. Notably, in combination with the chemotherapeutic cisplatin, prolonged survival and delayed recurrence were also reported [55]. Vistusertib is often used in combination treatment and has promising anti-cancer effects [57,58]. A phase 1b/2 trial (NCT02208375) investigated the maximum tolerated dose in patients with recurrent endometrial cancer and OC in combination with a PARP-inhibitor (results not published yet). Another recently published article described the efficacy and safety of vistusertib in combination with paclitaxel in a multicenter phase 2 trial. The authors indicated that the combination of vistusertib with paclitaxel did not improve the paclitaxel activity [59].

#### 3.1.4. Lurbinectedin

Lurbinectedin is an anti-tumor agent belonging to the family of tetrahydroisoquinoline and is structurally and functionally related to trabectedin [60]. The mechanism of action includes the formation of covalent bonds with guanines in specific nucleoside triplets located in the DNA, which can eventually lead to double-strand DNA breaks, cell-cycle arrest, and apoptosis of cancer cells [60]. Lurbinectedin has been approved for use as an orphan drug for the treatment of small-cell lung cancer, OC, and malignant mesothelioma through intravenous administration. In 2020, lurbinectedin was granted accelerated approval by the U.S. Food and Drug Administration (FDA) for the treatment of small-cell lung cancer patients after first-line chemotherapy [61,62]. The recommended dose is 3.2 mg/m^2^ body surface area (BSA). The most frequent adverse reactions include fatigue, pneumonia, dyspnea, respiratory infections, and musculoskeletal pain [61]. Interestingly, in OC, preclinical evidence has shown the effect of lurbinectedin on the immune microenvironment [63]. More specifically, the cytotoxic effect of lurbinectedin on human immune cells, including monocytes, was proven both in vitro and in vivo. In addition to the cytotoxic effects inducing the apoptosis of immune cells residing in the TME, a reduced production of inflammatory factors such as CCL2, CXCL8, and VEGF was observed, resulting in a diminished migration of tumor-promoting immune cells, including MDSC, to the TME [63]. Clinical trials were conducted in OC patients. One trial (NCT02421588) showed a good tolerability profile of the drug in patients with platinum-resistant disease. In this trial, a similar anti-tumor efficacy was reported as the standard of care pegylated liposomal doxorubicin (PLD) treatment, although the primary endpoint of improving the progression-free survival was not met [64]. A second trial (NCT01831089) testing lurbinectedin in combination with paclitaxel reported an acceptable safety profile and promising anti-tumor activity [65]. Lurbinectedin was administered as fixed doses ranging between 3.0 and 5.0 mg.

### 3.2. Candidate Treatments Inhibiting MDSC Immunosuppressive Functions

#### 3.2.1. Metformin

EMA-approved in 1958 and FDA-approved in 1994, metformin is a first-line therapy for patients with type 2 diabetes mellitus (T2DM). The mechanisms underlying the function of metformin are quite complex but are mostly driven by the activation of AMP-activated protein kinase [66]. It is used to prevent the onset of T2DM in at-risk patients and decreases the risk of complications associated with T2DM [67]. A typical posology is 1000 mg metformin as an oral formulation, with a target concentration ranging from 0.1 to 4 mg/mL [68]. Metformin shows a pharmacokinetic (PK) interaction with the anticancer compound vandetanib, where the coadministration of both compounds results in a change in metformin PK parameters, such as an increase in the area under the curve (AUC) and peak concentration (Cmax) [69]. The reported side effects of metformin include diarrhea, nausea, vomiting, and lactic acidosis (rare (1 in 30,000 patients) but severe) [70]. In OC patients suffering from T2DM, a prolonged survival was observed after metformin treatment compared to patients not receiving metformin [71]. Therefore, the dose of the metformin in this investigation was the same as for the original indication (T2DM). The mechanism possibly driving this clinical benefit is the impaired suppressive function caused by the inhibition of CD39/CD73 expression on MDSC after metformin treatment [71]. This clinical benefit could not be reproduced in another clinical trial in OC (NCT02312661), in which long-term metformin use could be identified as a favorable prognostic factor for overall mortality but not for the cancer-specific survival of OC patients [72].

#### 3.2.2. Celecoxib

Celecoxib is an approved COX2 inhibitor (EMA in 2000, FDA in 1998), originally indicated as a symptomatic relief in the treatment of osteoarthritis and rheumatoid arthritis with a recommended daily dose between 200 and 400 mg [73,74]. It has anti-inflammatory and analgesic effects and belongs to the family of non-steroidal anti-inflammatory drugs (NSAID). Drug–drug interactions are mainly ascribed to the cytochrome P450 (CYP) 2D6-mediated metabolism when combining celecoxib with inhibitors or inducers of this specific pathway [74]. Side effects include headache, diarrhea, abdominal discomfort, and dizziness [74]. In addition to its indicated use, celecoxib is deemed to be a potential cancer chemopreventive agent [75]. Interestingly, in OC, celecoxib-mediated COX2 blockade was observed to reverse the suppressive functions of MDSC in an in vitro patient-derived culture experiment [76]. This can be explained by the uncovered potentiation of MDSC suppression by type 1 inflammatory mediators such as IFN-gamma and TNF-alpha. The COX2 axis was further identified as the central downstream pathway regulating the immunosuppressive function of MDSC [76]. Furthermore, COX2 overexpression could be linked to an unfavorable prognosis in patients with OC. Therefore, a phase 2 clinical trial (NCT01124435) was conducted to test the efficacy of a combinational treatment of celecoxib with carboplatin. Patients were required to take 200 mg celecoxib twice daily by mouth for 28 days, in combination with intravenous carboplatin, which showed that the combinational treatment had a promising effect on overall survival and a tolerable safety profile [77].

#### 3.2.3. Nor-NOHA

Nor-NOHA or hydroxy-nor-1-arginine is an arginase-1 inhibitor currently under clinical investigation for the treatment of patients with coronary artery disease suffering from ischemia-reperfusion injury. The trial started because results showed promising effects on endothelial dysfunction in patients [78]. Similar results on microvascular endothelial function were observed in type 2 diabetes patients with microvascular dysfunction. In this study, no adverse effects were observed [79]. Preclinical studies in OC report an arginase-1-dependent suppression of CD8+ and CD4+ T cells by MDSC. The inhibition of arginase 1 using nor-NOHA subsequently restored the proliferative function of these T cell subsets in an in vitro assay performed by Baek and colleagues [80]. To our knowledge, nor-NOHA has not been tested in OC patients to date. However, another arginase inhibitor, INCB001158, has been tested in combination with chemotherapy in patients with solid tumors, including OC, in a phase 1/2 study (NCT03314935). Results from this study were only published for a subset of patients with advanced biliary tract cancers, showing good tolerability of the combination without added toxicity and promising response rates [81].

#### 3.2.4. Anti-CTLA4/CD80 mAb

Ipilimimab, an ICI targeting CTLA4, was the first ICI to be approved for the treatment of cancer [82]. In monotherapy, it is indicated for the treatment of advanced melanoma in adults and adolescents. In this indication, it is administered intravenously as an infusion with 3 mg/kg bodyweight every three weeks, for a total of four doses [83]. Side effects include diarrhea, rash, pruritis, fatigue, nausea, vomiting, decreased appetite, and abdominal pain [83]. Preclinically, the treatment of mice bearing OC with either anti-CTLA4 or its ligand CD80 was shown to slow down the tumor growth and reduce T-cell suppression by MDSC through direct cell–cell interaction. Both compounds were administered before and after tumor inoculation at a dose of 250 µg [84]. Although the immune checkpoint blockade of CTLA4/CD80 interaction showed promising results in preclinical mouse studies, this could not be reproduced in the clinic, similarly to the anti-PD-1 ICI. A phase 2 trial (NCT01611558) investigated the effect of ipilimumab (10 mg/kg) monotherapy in patients with platinum-sensitive OC. Of the 40 participants that were recruited for this trial, 10% showed the best response rate, defined as complete response/partial response. A total of 65% of the participants showed serious adverse events when treated with the investigated dosing regimen (11). Ipilimumab has also been tested in combination with nivolumab, a PD-1 inhibitor mAb, in a phase 2 trial (NCT02498600). The combinational treatment with ipilimumab showed a superior in treating patients with OC compared to nivolumab alone [85].

#### 3.2.5. Anti-PD-1/PD-L1 mAb

Anti-PD-1 or anti-PD-L1 refers to a group of ICIs targeting the interaction between the inhibitory checkpoint receptor PD-1 on immune cells and its ligand PD-L1. PD-1 inhibitors have been approved for the treatment of several types of cancer, including melanoma, lung cancer, bladder cancer, and kidney cancer. Approved anti-PD-1 mAbs include pembrolizumab, nivolumab, and cemiplimab [86]. Adverse events are mAb-specific and are commonly known as ICI-induced immune-related adverse events, with an occurrence of up to 70% [87]. Some of the most common side effects of checkpoint inhibitors are diarrhea, fatigue, cough, nausea, skin rash, poor appetite, constipation, muscle and joint pain [88]. Interestingly, the study by Liu et al. showed prolonged survival in a subcutaneous OC mouse model after the administration of a neutralizing mAb targeting either the receptor PD-1 or ligand PD-L1. The compound was administered intraperitoneally at a dose of 250 µg, before and after inoculation [89]. The prolonged survival can be linked to the role of PD-L1 in MDSC immunosuppression. In OC patients, the efficacy of anti-PD-1/PD-L1 blockade in monotherapy remains limited, as shown in multiple phase 1/2 trials [90]. Additionally, despite high hopes, combining treatment with an anti-PD-1 mAb with chemotherapy was unable to increase the success rate of this ICI in OC, as shown in multiple phase III trials [11,13]. However, other combinations with this ICI therapy might still result in a clinical benefit; for example, the combined treatment of anti-PD-1 and PARP inhibition, which is currently under investigation in several clinical trials (NCT03737643, NCT03602859, NCT03522246, NCT03598270, NCT03740165).

##### Combined Anti-PD-1 and GITR mAb

Anti-PD-1 can also be applied in combination with other treatments targeting immunosuppression by MDSC in OC. In the study performed by Lu et al., anti-PD-1 was combined with a mAb targeted towards the costimulatory receptor glucocorticoid-induced tumor necrosies factor receptor-related protein (GITR) [91]. GITR is upregulated on activated T cells, increasing their proliferation, activation and cytokine production [91]. Stimulation of this receptor using the anti-GITR mAb can enhance the anti-tumor activity of the immune system by inducing the expansion and activation of effector T cells and promoting the activity of other immune cells. Preclinical evidence demonstrates that a combined anti-PD-1/GITR treatment with 250 µg of each compound, when administered intraperitoneally, significantly inhibited tumor growth in tumor-bearing mice compared to either compound in monotherapy [91]. Notably, there was a significant shift from an immunosuppressive state to a more anti-tumor immune environment in the peritoneal cavity of these mice. In addition to an increase in effector CD8+ T cells, a significant reduction in MDSC was reported after combined therapy [91]. A phase 1 trial investigated the safety profile of TRX518, an anti-GITR mAb, in 43 patients. Patients received a single dose of TRX518 ranging from 0.0001 to 8 mg/kg bodyweight as an intravenous infusion. Sixteen patients experienced a treatment-related adverse event, of which the most common was fatigue. No dose-limiting adverse events occurred during the treatment with TRX518 [92]. The combination of anti-PD-1 (INCMGA0012) and GITR mAb (INCAGN01876) was tested in a phase 2 trial (NCT04225039), with and without radiosurgery in patients with recurrent glioblastoma. Anti-PD1 + anti-GITR has not been tested in a clinical trial for the treatment of OC to date.

##### Combined Anti-PD-1 and CD137 Activation

Guo and colleagues tested the combination of an agonistic anti-CD137 mAb with anti-PD-1 checkpoint inhibition in tumor-bearing mice. In this experiment, beneficial results were reported of an increased survival and a reversed immunosuppression due to fewer MDSCs being present after combination therapy [93]. Activation of the co-stimulatory receptor CD137 is an appealing target for the development of (combination) immunotherapy due to the preferential expression of this receptor on immune cells such as T-cells and NK cells, as well as oncogenic cells [94]. Two anti-CD137 mAb were investigated, urelumab, a human IgG4 mAb, and utomilumab, a humanized IgG2 mAb [94]. Some preliminary response was observed in lymphoma and Merkel cell carcinoma patients, but in the majority of cancers, results were disappointing [95]. In OC, a phase 1 trial (NCT03318900) included 18 patients with recurrent disease who were treated with utomilumab in combinational treatment with T cell infusion and aldesleukin (IL-2) (no results published). Another clinical trial is currently examining the effect of utomilumab with avelumab (NCT02554812). Overall, the results suggest that urelumab is safe at a dose of 8 mg or 0.1 mg/kg administered every 3 weeks [96], and utomilumab showed no dose-limiting toxicities [97]. However, the limited response could be related to MDSC lacking CD137 expression and thus evading the effect of this therapy, resulting in continued immunosuppression [98]. Therefore, combinations with ICI were tested to translate the efficacy of this therapy to the clinic.

#### 3.2.6. Combined Anti-TIM3 and CD137 Activation

The combination of the ICI anti-TIM3 with CD137 activation was tested by Guo et al., who administered a dose of 250 µg of each compound intraperitoneally and reported an increase in the CD8+ T cell/MDSC ratio in the peritoneal cavity of mice receiving combination therapy [99]. Anti-TIM3 mAb is an ICI targeted against the inhibitory T cell receptor immunoglobulin and mucin-domain-containing protein 3 (TIM3). The blockade of TIM3 can result in reduced tumor progression in preclinical models and can improve anti-tumor T cell responses in cancer patients [100]. Sabatolimab is a promising humanized mAb, which is currently being tested in clinical trials, mostly for the treatment of leukemia or myelodysplastic syndrome (NCT04623216 and NCT04812548). The safety and tolerability of another anti-TIM3 therapeutic (INCAGN0290) was tested in a phase 1 trial (NCT03652077) in cancer patients including OC, showing a linear PK with acceptable tolerability. The most frequently reported adverse events were anemia, fatigue, and back pain [101]. Another trial testing sabatolimab (NCT02608268) in a group of patients of which OC accounted for 17% concluded a similar tolerability profile, with fatigue as the most common reported adverse effect [102]. However, anti-TIM3 has not been investigated in combination with anti-CD137 in a clinical trial.

### 3.3. Candidate Treatments Depleting MDSC

#### 3.3.1. Dexamethasone

Dexamethasone belongs to the group of glucocorticoids and is widely used in the treatment of inflammatory disorders [103]. The use of dexamethasone experienced a revival in the beginning of the COVID-19 pandemic. At a time when therapeutic agents for a novel virus were urgently needed, the effect of dexamethasone on the clinical outcome of hospitalized patients with SARS-CoV-19 was extensively investigated [104,105,106,107]. The usual posology of dexamethasone is 40 mg orally once a day. PK interactions are mainly caused by the inhibition or induction of dexamethasone metabolizing enzymes such as CYP 3A4 [108]. Corticosteroids, such as dexamethasone, are well-described in the literature and are well-known from clinical practice. Known side effects include hypokalemia, myopathy, and glucose intolerance after short-term use, and osteoporosis, adrenal insufficiency, ophthalmologic side effects, and hyperlipidemia after long-term use [109]. Furthermore, it is indicated in adults for the treatment of symptomatic multiple myeloma in combinational treatment [110]. In an OC study by Lin et al., doses of 5, 50, and 100 µg/kg body weight were reported to reduce ovarian tumor growth and metastasis in tumor-bearing mice. Furthermore, it also decreased the expression of proinflammatory cytokines (IL-1beta and IL-18) and decreased MDSC and tumor-associated macrophage levels in the TME [111]. The exact mechanism through which dexamethasone influences MDSC is still unknown. In contrast, dexamethasone is commonly reported as an immune regulatory drug able to suppress the immune response in inflammatory diseases or transplantation [112]. However, it can be hypothesized that the decrease in inflammatory factors induced by dexamethasone treatment results in a lower MDSC, since a combination of specific cancer-derived transcription factors and inflammatory cytokines are required for the generation of MDSC in the bone marrow [42]. Dexamethasone is often explored in clinical trials in combination with chemotherapy to prevent nausea and vomiting. However, clinical evidence regarding the anticancer effect of dexamethasone in patients with OC is lacking.

#### 3.3.2. CXCR4 Antagonist, Plerixafor, Combined with Anti-PD-1

FDA-approved in 2008, and EMA-approved one year later, plerixafor is a CXCR4 antagonist indicated for the treatment of patients with lymphoma or multiple myeloma. It is administered subcutaneously at a dose of 0.24 mg/kg bodyweight. Plerixafor-associated side effects include gastrointestinal adverse events such as diarrhea and nausea and injection-site reactions of erythema and pruritis [113,114]. In cancer, the CXCR4 pathway, induced by the binding of its ligand CXCL12, was identified to promote metastasis and angiogenesis, and to regulate immune dysfunction [115]. In preclinical OC research, plerixafor treatment with a dose of 3 mg/kg bodyweight could prolong survival in a metastatic OC mouse model. The combination of plerixafor and anti-PD-1 ICI showed an additional survival benefit compared to plerixafor alone in a study by Zeng et al. Additionally, it was shown that this combination treatment had an indirect effect on the MDSC by decreasing their abundance in the TME of treated mice, which was not seen in mice treated with plerixafor only [116]. The expression of CXCR4 on ovarian cancer cells was associated with an unfavorable outcome for patients with reduced progression-free and overall survival [115]. A phase 1 trial (NCT02179970) aimed to assess the safety of the continuous intravenous administration of plerixafor in patients with pancreatic, ovarian, and colorectal cancer. Although no results have been published for OC, in pancreatic and colorectal patients, an increased anti-tumor immune response was observed in plerixafor-treated patients. Treatment was well-tolerated after infusion at a dose rate of 80 µg/kg per hour and no dose-limiting toxicities were reported [117].

#### 3.3.3. Dabigatran Combined with Cisplatin

Dabigatran is an oral anticoagulant therapeutic agent with EMA approval in 2008 and FDA approval in 2010. It belongs to the family of reversible direct thrombin inhibitors [118,119], interacts with the coagulation cascade and subsequently prevents the development of a thrombus [120]. Therefore, it is used in the prevention and treatment of venous and arterial thrombosis [118]. The most common side effect of dabigatran is bleeding. Dabigatran cannot be used in patients suffering from reduced kidney function, liver or heart problems [119]. For more a century, there has been evidence of a clinical correlation between thrombosis and cancer. The expression of coagulation factors and indicators of hematostatic system activation are highly correlated, resulting in a poor prognosis for many types of cancer [121,122,123]. Therefore, the combined administration of dabigatran and cisplatin was hypothesized to enhance anti-tumor efficacy. In OC, Alexander et al. investigated the effect of oral dabigatran 80 mg/kg, in combination with intraperitoneal cisplatin 1 mg/kg, in tumor-bearing mice. The results showed reduced ascites’ formation and decreased tumor growth [124]. Furthermore, in a later study, the addition of the ICI anti-PD-1 to the cisplatin/dabigatran combination was able to prolong survival in the same OC mouse model [125]. Interestingly, sa ignificant depletion of MDSC was unexpectedly discovered in the ascites of a metastatic mouse model after the co-treatment of dabigatran and low-dose cisplatin [124]. Although cisplatin is widely used for the treatment of OC, combination therapy with dabigatran has yet to be tested in the clinic.

### 3.4. Candidate Treatments Able to Reprogram MDSC

#### 3.4.1. 5-Azacytidine

5-azacytidine is a pyrimidine nucleoside analog functioning as DNA methyltransferase inhibitor. This hypomethylating agent was approved for the treatment of myelodysplastic syndromes such as myeloid leukemia in 2004 [126,127]. For this indication, a treatment cycle consists of seven days of the subcutaneous administration of 75 mg 5-azacytidine/m^2^ body surface area, followed by a 21-day rest period. 5-azacytidine is well-described in the literature, with a long history of clinical experience. Very common side effects include anemia, gastrointestinal disorders, pneumonia, fatigue, headache, and rash [128]. In OC, 5-azacytidine prolonged survival and reduced ascites’ production in tumor-bearing mice. In addition to the direct anti-tumorigenic effect of 5-azacytidine, a reduction in the number of MDSC in the ascites fluid was reported, which could be related to the activation of type I interferon signaling [129]. Increased type 1 interferon signaling has been suggested to lower MDSC activation through reprogramming [130]. In a phase 1 trial (NCT00529022), 32 patients with advanced solid tumors, of which 10 patients had platinum-refractory or resistant OC, were recruited and the effects of the combination treatment of 5-azacytidine (s.c.) with valproic acid and carboplatin was investigated. In six patients, minor responses or stable disease were achieved. The most common side effects were fatigue, and neutropenia, and dose-limiting adverse side effects occurred in six patients [131]. Additionally, a clinical trial was conducted in which the effect of oral 5-azacytidine was tested in combination with pembrolizumab (NCT02900560) in patients with platinum-resistant OC. Patients received 100–300 mg once or twice daily for two or three weeks, in combination with 200 mg intravenous pembrolizumab every 21 days (no results published).

#### 3.4.2. Anti-IL-10 Receptor mAb

The anti-inflammatory cytokine IL-10 has been considered a potential therapeutic target and tested in different clinical trials for multiple diseases such as cancer, autoimmune, and neurodegenerative diseases [132,133]. For example, the neutralizing humanized anti-IL-10 mAb, BT063, is being investigated in a phase 2 trial (NCT02554019) for the treatment of systemic lupus erythematosus (SLE). B-N10, a second anti-IL-10 mAb, was also tested in six patients with SLE, receiving 20 mg/day for 21 consecutive days. The authors reported one single adverse event for one patient, who experienced a weak adverse event, consisting of chills, during the B-N10 infusion [134]. The blockade of IL-10 signaling succeeded in lifting immunosuppression by MDSC and improved survival in an OC mouse model in a study by Hart and colleagues [135]. MDSC were previously observed to require IL-10 for the development and maintenance of their immunosuppressive function [136]. In OC patients, blocking IL-10 has not been investigated to date.

## 4. Discussion

**Figure 3:**
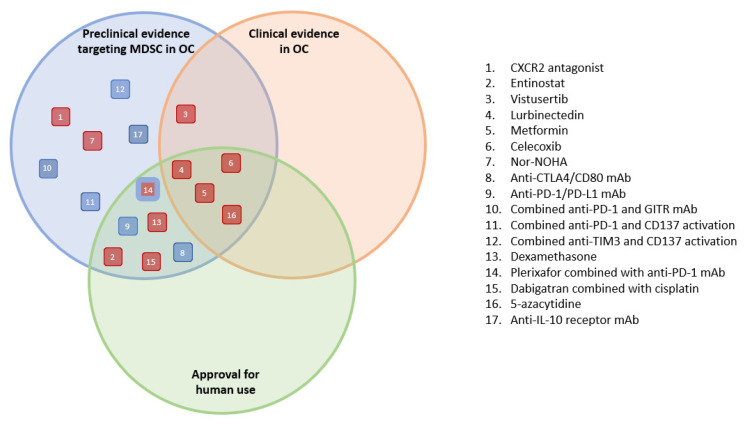
Overview of the evidence of candidate drugs for the treatment of patients with ovarian cancer. Approval for human use relates to an indication outside of ovarian cancer. The symbols are color-coded; red and blue symbols correspond to small molecules and protein-based compounds, respectively. OC, ovarian cancer; MDSC, myeloid-derived suppressor cells.

This review provides an overview of possible candidate drugs targeting MDSC in the TME of OC. For each candidate, the original use or indication and known side effects are given, as well as the preclinical and clinical evidence for repurposing, aiming to block MDSC immunosuppression in OC. Figure 3 provides an overview of all candidate drugs, classified by their available information/evidence for the treatment of patients with OC.

From Figure 3, we can conclude that four compounds have the most evidence for repurposing, with both preclinical and clinical evidence in OC as well as previous approval for human use in another indication. These candidate drugs include lurbinectedin, metformin, celecoxib, and 5-azacytidine. The first drug, lurbinectedin, shows great promise for repurposing, with a previously demonstrated anti-tumor response in OC patients and an acceptable safety profile. The effect on MDSC has only been proven in preclinical studies, although it is also suggestive of beneficial clinical results. These results are supported by further research focusing on chronic lymphocytic leukemia, where lurbinectedin was observed to significantly decrease MDSC percentages in in vitro patient-derived studies [137]. Metformin is the second compound we would like to highlight in this article. Two clinical trials led to ambiguous results with contrary clinical outcomes, which might be explained by the different settings, dosing regimens, study size, or study duration. Metformin is well-described in the literature regarding its PK profile, with in-depth clinical experience. It comes with known side effects and contraindications that need careful consideration when treating patients. However, the depth of clinical experience and the overall good tolerance of the drug compared to anticancer drugs still make it a valid candidate for MIDR approaches and clinical trial simulations to guide trial designs in patients with OC and further identify patient subgroups that do not benefit from the treatment. The third promising candidate, celecoxib, presents as an auspicious repurposing candidate for targeting MDSC in OC because of the anti-inflammatory properties, which would potentially lower the MDSC immunosuppressive function. Moreover, tolerability has been confirmed in OC patients. In a mesothelioma mouse model, this drug similarly prevented MDSC proliferation and impaired the immunosuppressive function, suggesting that celecoxib can provide a benefit when used in combination with immunotherapy approaches [138]. Lastly, 5-azacytidine has shown promising preclinical results in OC, both alone and in combination with entinostat therapy. Moreover, in preclinical lung and adenocarcinoma mouse models, Mikyskova et al. demonstrated a depletion of the MDSC immunosuppressive function [139]. Although 5-azacytidine has not resulted in significant survival improvements for OC patients in clinical trials to date, stable disease and a minor benefit were reported. One limitation of this treatment is the occurrence of adverse effects. However, because of the extensive clinical experience, these side effects can be taken into consideration when repurposing this drug and designing the optimal treatment regimen for each patient. Metformin and celecoxib have shown positive effects in the treatment of OC when administering an approved dose as for its original indication. The dosing of lurbinectedin and 5-azacytidine differs from its original indication. However, assuming that the optimal dose, which strikes the right balance between effectiveness and safety for one indication, is also the optimal dose for another indication, is risky. As stated by the EMA, agencies should be open to the use of statistical and pharmacometrics techniques such as Bayesian and population methods when it comes to dose-finding [140,141]. It is crucial to further explore the dose–exposure–response relationship of these compounds across a broader dosing regimen, instead of looking at a single dose [141]. Model-informed approaches can be a useful way to explore this relationship and develop tailored dosing regimens for each indication.

During our literature search, another promising therapeutic target was discovered. Because preclinical experiments were not performed in OC, this article was not included in Figure 2. From an OC database screening, Capping Actin Protein, Gelsolin-Like (CAPG) gene expression could be correlated to a rise in MDSC levels in the TME of OC. Based on this information, the authors looked at drug databases to select five candidate drugs through in situ analysis that could target this overexpression-induced pathway: valproic acid, JQ1, tretinoin, vorinostat, and vitamin E1. Although no proven evidence exists at this moment, these drugs represent promising candidates for repurposing towards MDSC targeting in OC [142].

We acknowledge some limitations of our original literature research. The literature research was restricted to English articles. Therefore, articles published in a different language were not included and are not part of this review. We focused on one database to identify articles; additional articles that were available on different platforms could have been missed. Furthermore, the use of a standardized template to extract relevant information required sufficient information regarding the experiment/trial, the investigated compound, and the outcome. Study protocols, studies with insufficient information in the article, and ambiguous results could, therefore, not be used, and, consequently, these articles are not part of this review. Another limitation could be the heterogeneity of different cancer patients possibly annulling the positive effect of the repurposed drugs in some patients. Therefore, even for repurposed treatments, the use of biomarkers, genetics, and personalized medicine (including personalized dose optimization) could provide assistance in the future [34]. Nevertheless, our focus on preclinical studies while identifying suitable candidates enables an early insight into potential repurposable drugs and their possible immunological effect on the OC drug development process.

## 5. Conclusions

The repurposing of drugs can be a promising approach when designing new treatment strategies. Since MDSC are known to suppress the immune response in OC using many different mechanisms, finding an optimal candidate drug to target their immunosuppressive function is challenging. Therefore, we believe that the repurposing of drugs can offer timely opportunities. Here, we discussed multiple repurposed drug candidates and combination treatments that have shown promising results in preclinical studies for targeting MDSC in OC, some of which have shown clinical evidence in OC. The main challenge is to further test the repurposed candidates, find their specific indication and explore combinations with other (immune-)therapies.

## Data Availability

No new data was created for this review.

## Supplementary Materials

The following supporting information can be downloaded at: https://www.mdpi.com/article/10.3390/pharmaceutics15071792/s1, Supplementary Material S1: final search term pubmed; Supplementary Figure S1: Chemical structure of the included compounds.
