# Supplementary material for: Drug Repurposing for Targeting Myeloid-Derived Suppressor-Cell-Generated Immunosuppression in Ovarian Cancer: A Literature Review of Potential Candidates
Source: Pharmaceutics. 2023 Jun 22;15(7):1792. doi: 10.3390/pharmaceutics15071792 (PMC10385967; doi:10.3390/pharmaceutics15071792)
Supplement: Supplementary file 1 [file pharmaceutics-15-01792-s001.zip › pharmaceutics-2415351-supplementary.pdf]

pharmaceutics

Supplementary Materials: Drug Repurposing for Targeting Myeloid-Derived Suppressor Cell Generated Immunosuppression in Ovarian Cancer: A Literature Review of Potential Candidates

Yani Berckmans, Yannick Hoffert, Ann Vankerckhoven, Erwin Dreesen, An Coosemans

Suplementary materials S1: final search term pubmed

("Ovarian Neoplasms"[MeSH] OR (ovar* [tiab] AND (tumor* OR tumour* OR cancer* OR carcinoma* OR malignan* OR neoplasm* [tiab]))) AND (((Myeloid-Derived Suppressor Cells[MeSH Terms] OR myeloid-derived suppressor*[tiab] OR Granulocyte Precursor Cells[MeSH Terms] OR Granulocyte precursor*[tiab] OR Monocyte-Macrophage Precursor Cells[MeSH Terms] OR Monocyte-Macrophage Precursor*[tiab] OR Myeloid Progenitor Cells[MeSH Terms] OR Myeloid Progenitor*[tiab] OR MDSC*[tiab] OR myeloid suppressor*[tiab] OR MSC[tiab] OR MSCs[tiab] OR natural suppressor*[tiab] OR null cell*[tiab] OR immature myeloid cell*[tiab] OR IMC[tiab] OR N2 neutrophil*[tiab] OR (Myeloid[tiab] AND deriv*[tiab]) OR (Myeloid[tiab] AND suppress*[tiab]))) OR ((CD11b[tiab] OR CD11b Antigen[MeSH Terms]) AND (GR1[tiab] OR Ly6G[tiab] OR Ly-6G[tiab] OR Ly6G antigen, mouse[MeSH Terms] OR Ly6C[tiab] OR Ly-6C antigen, mouse[MeSH Terms])) OR ((CD11b[tiab] OR CD11b Antigen[MeSH Terms]) AND (CD33[tiab] OR Sialic Acid Binding Ig-like Lectin 3[MeSH Terms]) AND (CD14[tiab] OR Lipopolysaccharide Receptors[mesh Terms] OR CD15[tiab] OR Lewis X Antigen[MeSH Terms] OR HLA-DR[tiab] OR HLA-DR Antigens[MeSH Terms])))

Resulting in 605 articles on 26/04/2022

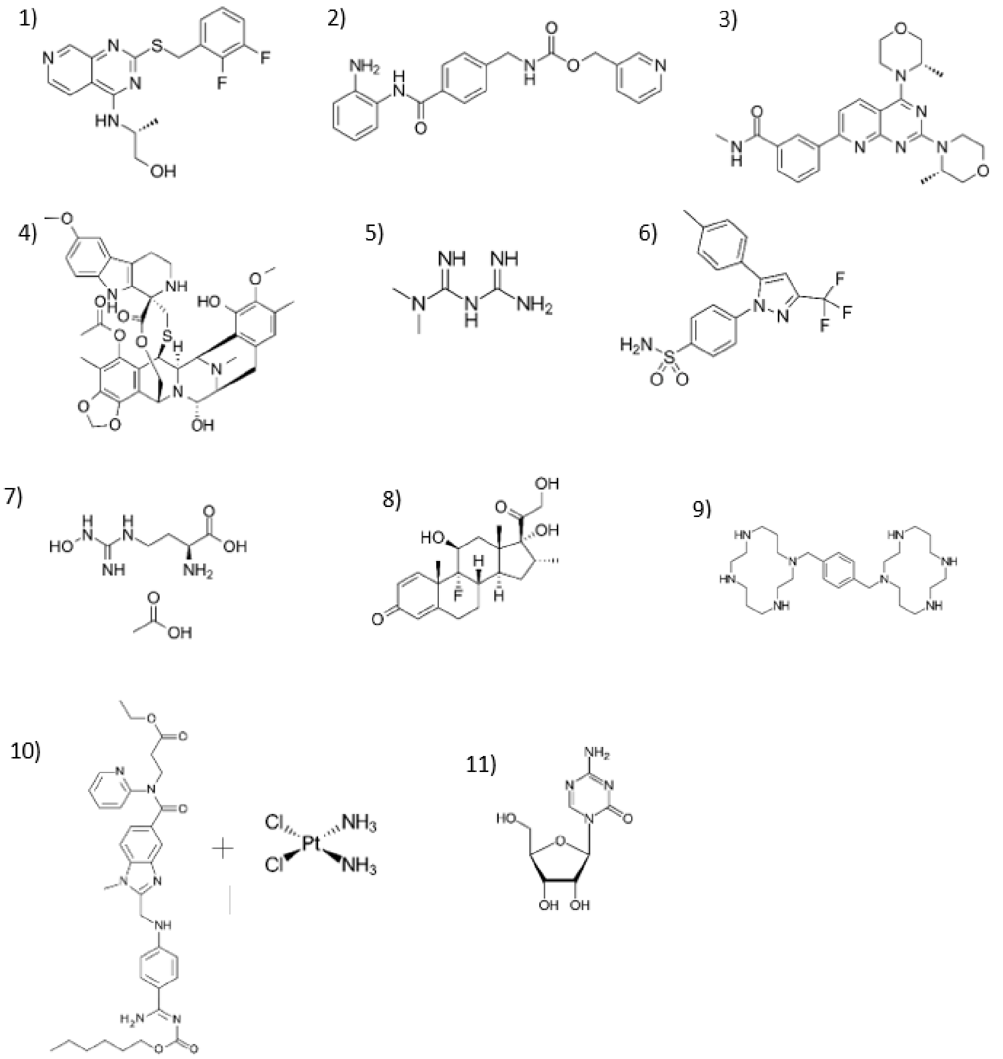

Figure S1. Chemical structure of the included compounds. 1) CXCR2 antagonist; 2) entinostat; 3) vistusertib; 4) lurbinectedin ; 5) metformin; 6) celecoxib; 7) nor-NOHA; 8) dexamethasone; 9) plerixafor (left) combined with anti-PD-1 (right; general schematic structure); 10) dabigatran (left) and cisplatin (right); 11) 5-azacytidine. Structures of monoclonal antibodies are not included.
